# The feasibility and acceptability of home phlebotomy for patients with cancer

**DOI:** 10.1093/jncics/pkae104

**Published:** 2024-10-16

**Authors:** Erin M Bange, Camila Bernal, Kemi Bolutayo Gaffney, Jill Ackerman, David Kwong, Jithin Thomas, Bobby Daly

**Affiliations:** Department of Medicine, Memorial Sloan Kettering Cancer Center, New York, NY 10021, United States; Department of Medicine, Memorial Sloan Kettering Cancer Center, New York, NY 10021, United States; Department of Medicine, Memorial Sloan Kettering Cancer Center, New York, NY 10021, United States; Department of Medicine, Memorial Sloan Kettering Cancer Center, New York, NY 10021, United States; Department of Medicine, Memorial Sloan Kettering Cancer Center, New York, NY 10021, United States; Department of Medicine, Memorial Sloan Kettering Cancer Center, New York, NY 10021, United States; Department of Medicine, Memorial Sloan Kettering Cancer Center, New York, NY 10021, United States

## Abstract

Time toxicity is a considerable burden for oncology patients. This study evaluated the feasibility and acceptability of integrating mobile phlebotomy into standard of care procedures. From September 26, 2022, through December 31, 2023, a total of 345 patients had 1464 home laboratory test collection visits completed. These mobile phlebotomy laboratory collection visits occurred in New York (68.6% of visits), New Jersey (29.9%), Connecticut (1.1%), and Pennsylvania (0.5%). Specimen quality for home laboratory test collection surpassed the Memorial Sloan Kettering Department of Pathology and Laboratory Medicine benchmarks. Acceptability was high, 173 patients were approached, and 149 responded (86% response rate); most respondents (147 of 149, 99%) would use the service again or recommend it to others. This study assessed the integration of mobile phlebotomy into standard of care management for the collection of routine cancer laboratory tests. Mobile phlebotomy results in high patient satisfaction with superior specimen quality, offering a valuable solution to oncology patients for improved efficiency and convenience.

## Introduction

Individuals with cancer typically dedicate an average of 16 hours per month over a 4-5 day period to activities related to their anticancer treatment^1^ of which more than 50% of that time is spent commuting or waiting for care.^2^ Research to mitigate time toxicity has primarily focused on strategies to optimize clinic function through improved workflows,^3^ space utilization,^3^ and provider availability.^4^ However, there is a growing interest to address time toxicity by providing more care at home of which mobile phlebotomy is an essential step. For mobile phlebotomy services to be integrated into standard of care procedures, collection and reporting of specimens must be on par with in-clinic phlebotomy services. In this study, we tested the feasibility and acceptability of integrating a mobile phlebotomy service into standard of care laboratory procedures at the Memorial Sloan Kettering Cancer Center (MSK).

## Methods

Patients who received care at MSK, which comprises the New York City campus as well as the Regional Care Network Sites in New Jersey, Westchester, and Long Island, lived within the mobile phlebotomy catchment area, and were amenable to a venipuncture were eligible for inclusion. The mobile phlebotomy workflow was embedded into our standard of care procedures such that laboratory test ordering and result reporting were completed through the electronic medical record (Table S1). Once orders were placed by the provider, we partnered with a mobile phlebotomy company to schedule and coordinate at-home specimen collection. The laboratory specimen was then delivered by the mobile phlebotomy vendor to an MSK laboratory for processing. The results appeared in the electronic medical record in the same manner as those drawn onsite at MSK.

Feasibility was assessed using key performance indicators and quality standards that were set a priori by the MSK Department of Pathology and Laboratory Medicine.^5^^,^^6^ Data regarding specimen quality was collected from September 26, 2022, through December 31, 2023. Sex and ethnicity are self-reported and were extracted from the electronic medical record.

All patients who completed at least 1 at-home phlebotomy visit between September 26, 2022, and March 15, 2023, were called and offered a brief survey to measure acceptability. Patients were prompted if they would be willing to use this service again or recommend it to another patient with a yes or no response. This study received a waiver of informed consent from the institutional review board as a quality improvement study.

## Results

From September 26, 2022, through December 31, 2023, a total of 1464 home laboratory collection visits were completed for 345 patients (Table 1) in New York (n = 1004 [68.6%] visits), New Jersey (n = 437 [29.9%] visits), Connecticut (n = 16 [1.1%] visits), and Pennsylvania (n = 7 [0.5%] visits). The specimen quality for home laboratory test collection (Table 2) surpassed MSK Department of Pathology and Laboratory Medicine benchmarks. Of the 5104 samples collected (Table 2), only 1 (0.02%) specimen clotted, zero specimens were hemolyzed, 1 (0.02%) specimen was unlabeled, and 245 (4.80%) specimens had a turnaround time greater than 120 minutes (no impact to specimen quality). There were no cancellations because of sample stability, contamination, or other collection issues.

**Table 1.** myOnsite sociodemographic information

Age		
Median (range), y	69 (20-95)	
Sex, No. (%)		
Female	124	35.94
Male	221	64.06
Grand total	345	
Ethnicity, No. (%)		
Hispanic	27	7.83
Non-Hispanic	292	84.64
Unknown	26	7.53
Grand total	345	
Race, No. (%)		
Asian-Far East, Indian subcontinent	13	3.77
Black or African American	35	10.14
Other	12	3.48
Patient refused to answer, unknown	20	5.79
White, No	265	76.81
Grand total	345	
Marital status, No. (%)		
Married	231	66.96
Single	47	13.62
Widowed	30	8.70
Divorced	26	7.54
Partnered	6	1.74
Unknown	3	0.87
Separated	2	0.58
Grand total	345	

**Table 2.** Mobile phlebotomy specimen collection quality^a^

Performance indicator	Benchmark	Home laboratory collection visits (n = 1464)	Laboratory samples collected	Outlier, actual	Outlier, %
Clotted	2%	1464	5104	1	0.02
Hemolyzed	1%	1464	5104	0	0.00
Mislabeled	0	1464	5104	0	0.00
Unlabeled	0	1464	5104	1	0.02
Quantity not sufficient	2%	1464	5104	0	0.00
Wrong container	0	1464	5104	0	0.00
Turnaround time, collect to received^b^	120 minutes from collect to receipt at Memorial Sloan Kettering Cancer Center lab	1464	5104	245	4.80
Cancellations (due to sample stability, possible contamination, or other collection deficiencies)	0	1464	5104	0	0.00

a Data collection is from September 26, 2022, through December 31, 2023.

b The turnaround time was set at 120 minutes by the Department of Pathology and Laboratory Medicine. For the 245 specimens that had a turnaround time greater than 120 minutes, there was no impact on specimen quality.

In terms of acceptability, 173 patients were approached, and 149 responded (86% response rate). Most respondents (147 of 149, 99%) reported that they would use the service again or recommend the service to others.

## Discussion

This study examines the integration of mobile phlebotomy into standard of care procedures at a National Cancer Institute Comprehensive Cancer Center for the collection of routine cancer laboratory tests demonstrating that mobile phlebotomy is feasible and acceptable to patients with cancer. Mobile phlebotomy provides an opportunity to reduce the time toxicity for oncology patients, which is increasingly recognized as a substantial burden in need of solutions.^7^ Mobile phlebotomy addresses time toxicity in the following ways:

1. It optimizes the patient’s schedule; the sample can be drawn at the patient’s home or work in minutes saving the patient the clinic travel and wait times.
2. By collecting laboratory specimens at home prior to treatment, a patient’s laboratory results can be compared against the treatment parameters prior to the patient arriving at clinic, and if a value is outside of parameters, then treatment can be modified or rescheduled. This saves the patient the considerable stress and time of having to commute to clinic only to be turned away for results outside of parameters. As one patient stated: “Having blood work done the day before treatment at home is actually great. (1) It cuts the back-up at the clinic because you can review and approve it the day before so there is no delay in getting treatment ready when you check in, and (2) if there is a problem, it can be identified and hopefully fixed so that the treatment is not delayed.”

The benefits, however, need to be weighed against the institutional cost of implementing mobile phlebotomy because, for the majority of patients, it is not reimbursed by insurance. In this study, the cost of the mobile phlebotomy was covered by the institution although the laboratory processing was reimbursed through standard insurance mechanisms. In analyzing the return on investment of mobile phlebotomy, health-care leaders must consider the following:

1. Institutional savings through improved efficiency: By shifting laboratory collection to the home, leaders have the opportunity to increase efficiency in the infusion center. The National Comprehensive Cancer Network Infusion Efficiency Workgroup Study found the average patient wait time in infusion centers ranged from 25 to 102 minutes,^8^ and laboratory collection is a critical variable in determining these wait times.^9^ Long wait time leads to decreased patient satisfaction and increased institutional cost in overtime staffing. In addition, the efficient use of the infusion unit is compromised as more patients could potentially be treated if wait times were minimized and patients with laboratory results not meeting parameters were rescheduled prior to their clinic appointment. A positive return on investment for mobile phlebotomy could thus be achieved through the efficient delivery of more antineoplastic infusions by optimizing for the variable of laboratory collection and result analysis.
2. High patient satisfaction: The study demonstrated high patient satisfaction with mobile phlebotomy, which can translate to improved patient loyalty and serve as a differentiator for the institution in the marketplace. Indeed, the high net promoter score for mobile phlebotomy may itself contribute some financial value as patient experience metrics are associated with increased profitability.^10^^,^^11^
3. Shift toward convenience in health care: Relatedly, patients are increasingly prioritizing proximity and convenience in their choice of clinicians. Characterized as the “practical patient,” these individuals expect health-care services “to be readily available with minimal wait times and convenient locations.”^12^ According to the 2024 McKinsey Healthcare Report, consumers are particularly open to innovative care models that would allow for more care management at home^13^ and may be willing to pay an additional fee for this convenience. The emphasis on convenience is especially true for younger generations that are accustomed to on-demand services, and institutions that don’t adopt models that incorporate convenience do so at their own risk.^14^^,^^13^
4. Infrastructure for decentralized and remote care: In addition to routine lab collection at home for standard of care therapies, other components of cancer care are shifting to the home in the post pandemic era.^15^ There has been a shift toward decentralized clinical trial models where laboratory collection, imaging, symptom monitoring, and even investigational therapeutics are being provided closer to home.^16^^,^^17^ By building the capacity to leverage laboratory collection at home, institutions position themselves well to participate in these innovative care delivery models and develop the infrastructure to provide other services in the home.
5. Build vs buy and considerations of scale: In deciding on the implementation investment, institutions will also need to carefully consider whether to build a mobile phlebotomy service of their own vs contract with an outside vendor. If an institution chooses to contract with an outside vendor, it might lose control of quality, though this was not seen in our study. However, it could achieve lower per patient costs as the vendor may serve more patients leveraging economies of scale with multiple clients. For example, another institution that built its own mobile phlebotomy service reported only serving an average of 5 visits per day.^18^

This study is limited in that it occurred at 1 National Comprehensive Cancer Center; however, it did include our regional network sites and served a diverse population of patients across 4 states. In addition, the study collaborated with 1 mobile phlebotomy vendor to perform this intervention, and quality standards may vary by vendor selection.

In conclusion, increasingly, mobile phlebotomy is being considered for patients with cancer to improve access to standard of care and investigational treatments by reducing time toxicity by providing care in the home. Sustainability of such services will depend on adequate vendor integration into standard of care procedures and considerations of the return on investment, such as institutional savings resulting from more efficient use of clinic site resources and adoption of new care delivery models that respond to patient demand for convenience.

## Supplementary Material

pkae104_Supplementary_Data

## Data Availability

Data will be made available upon reasonable request to the corresponding author.
