# Supplementary material for: The feasibility and acceptability of home phlebotomy for patients with cancer
Source: JNCI Cancer Spectr. 2024 Oct 16;8(6):pkae104. doi: 10.1093/jncics/pkae104 (PMC11547947; doi:10.1093/jncics/pkae104)
Supplement: pkae104_Supplementary_Data [file pkae104_supplementary_data.docx]

Supplementary Material

| Supplementary Table 1. Laboratory tests eligible for home phlebotomy collection | | |
| --- | --- | --- |
| Alpha fetoprotein (AFP)​ | Activated Partial Thromboplastin Time (APTT)​ | Amylase Plasma |
| Amylase Total | Basic Metabolic Panel​ | Cancer Antigen 125 (CA 125)​ |
| Cancer Antigen 15-3 (CA 15-3)​ | Cancer Antigen 19-9 (CA 19-9)​ | Calcium​ |
| Complete Blood Count (CBC), Standard​ | Complete Blood Count (CBC), with ANC only​ | Complete Blood Count (CBC) WITH Differential​ |
| Carcinoembryonic Antigen (CEA)​ | Comprehensive Metabolic Panel (CMP)​ | CMP and Direct Bilirubin |
| Cortisol (0 min) | COVID-19 Spike Protein Antibody IgG​ | Creatinine​ |
| Electrolytes​ | Estradiol | Ferritin​ |
| Free Light Chains, Serum​ | FSH (Follicle-stimulating hormone) | Glucose​ |
| Growth Hormone | Pregnancy Test, Blood​ | Hemoglobin A1c (HGB-A1c)​ |
| Hepatic Function Panel Plasma | Hepatitis B Virus Core Antibody, Total​ | Hepatitis B Virus Surface Antibody​ |
| Hepatitis B Surface Antigen​ | Hepatitis C Virus Antibody​ | IgG / IgA / IgM​ |
| Immunofixation, Blood​ | Lactate Dehydrogenase (LDH)​ | Lipase​ |
| Lipid Panel​ | LH (Luteinizing Hormone) | Magnesium​ |
| Osmolality, Serum | Prolactin | Phosphorus​ |
| Prostatic Acid Phosphatase, Serum​ | Protein Electrophoresis​ | Prostate Specific Antigen (PSA)​ |
| PSA/Free PSA-​ | Prothrombin Time (PT), International Normalized Ratio (INR)​ | Thyroxine (T4), Free​ |
| Testosterone​ | Thyroglobulin Antibody​ | Protein, Total​ |
| Thyroid Stimulating Hormone (TSH)​ | Uric Acid​ | Urinalysis, Routine​ |
| Vitamin B-12 Level​ | Vitamin D 25 Hydroxy​ |  |
